# Folic Acid Alleviates Hydrogen Peroxide-Induced Oxidative Stress in Bovine Placental Trophoblast Cells by Regulating the NRF2/mTOR Signaling Pathway

**DOI:** 10.3390/ijms26062818

**Published:** 2025-03-20

**Authors:** Liyuan Shi, Zhisheng Wang, Jianxin Xiao, Rui Hu, Huawei Zou, Junmei Wang, Ziqi Yue, Quanhui Peng, Yahui Jiang, Bai Xue, Lizhi Wang

**Affiliations:** Low Carbon Breeding Cattle and Safety Production University Key Laboratory of Sichuan Province, Animal Nutrition Institute, Sichuan Agricultural University, Chengdu 611130, China; sly2021214030@126.com (L.S.); xiaojianxin@sicau.edu.cn (J.X.); zhwbabarla@126.com (H.Z.); junmeiwangsicau@163.com (J.W.); yzq947036981@163.com (Z.Y.); pengquanhui@126.com (Q.P.); jiangyahui.ff@163.com (Y.J.); xuebai@sicau.edu.cn (B.X.); 1285@sicau.edu.cn (L.W.)

**Keywords:** bovine placental trophoblast cells, oxidative stress, folic acid, NRF2/mTOR signaling pathway

## Abstract

As one of the important components of placental structure, the integrity of placental trophoblast cells is crucial for placental function. When oxidative stress continues to act on placental trophoblast cells, it can cause changes in placental structure and function. Research has shown that folic acid (FA) has a certain alleviating effect on the functional damage of trophoblast cells caused by oxidative stress, but the mechanism of action is still unclear. Therefore, this study focuses on bovine placental trophoblast cells (BPTCs) to explore the effects and mechanisms by which FA regulates oxidative stress in cells, with the aim of providing a theoretical foundation for improving the reproductive performance of cows. The results show that, compared with the H_2_O_2_ group, the FA+ H_2_O_2_ group showed an increase in the cell proliferation index (PI), superoxide dismutase 2 (*SOD2*), glutathione peroxidase (*GSH-px*), and catalase (*CAT*) mRNA expression and total antioxidant capacity (T-AOC) of cells, while the content of reactive oxygen species (ROS) decreased. In addition, the mRNA expression of tight junction factors, nutrient transporters, placental functional factors, mammalian rapamycin (mTOR) and its downstream factors, and nuclear factor erythroid 2-related factor 2 (NRF2) and its downstream factors in the FA+ H_2_O_2_ group increased, while the protein abundance of nuclear NRF2 decreased. After treatment with the inhibitor ML385, it was found that the protective effect of FA on H_2_O_2_-induced cellular oxidative damage was alleviated. These results indicate that FA can regulate the NRF2/mTOR signaling pathway, promote the expression of antioxidant factors, and alleviate the damage to the cell barrier and nutrient transport function in BPTCs caused by oxidative stress.

## 1. Introduction

Pregnancy disrupts the physiological balance of cows due to the presence of the fetus. During the transition period, cows are prone to negative energy balance (NEB), which can lead to maternal oxidative stress [1,2]. Research has shown that placental trophoblast cells are particularly vulnerable to oxidative damage caused by reactive oxygen species (ROS) [3]. Oxidative stress can adversely affect cell proliferation [4], apoptosis [5], and immunity [6], resulting in alterations to the structure and function of the placenta. The placenta plays a vital role in nutrient transport between the mother and fetus, which is essential for maintaining a healthy pregnancy and promoting fetal growth and development. Trophoblast cells serve as the fundamental structural units of the placenta, responsible for nutrient transport and the integrity of tight junctions [7,8]. The placental barrier is a critical component of maternal–fetal interaction, regulating, allocating, and even synthesizing vital nutrients, hormones, and growth factors necessary for fetal development and pregnancy maintenance. It also removes metabolic waste from fetal circulation and limits fetal exposure to toxins [9,10]. Studies have confirmed that the tight junction function of placental trophoblast cells is essential for the development of placental barrier integrity [11]. When trophoblast cells are damaged by excessive ROS oxidation, the integrity of the placental tight junctions is compromised, adversely affecting placental barrier function and, ultimately, fetal growth and development. Therefore, it is imperative to prevent oxidative stress in cows during pregnancy to mitigate placental damage.

Folic acid (FA), a member of the vitamin B9 family, is a water-soluble B vitamin with several important biological properties. It is an essential component for the development of the fetal brain and spinal cord. The neural tube, which forms the brain and spinal cord, develops early in pregnancy, and adequate intake of FA during this critical period can significantly reduce the risk of neural tube defects (NTDs) [12]. As the fetus undergoes rapid growth and development, FA plays a crucial role in DNA synthesis, repair, and methylation, producing nucleotides that are vital for cell division and tissue growth [13]. Sufficient maternal FA levels during pregnancy can prevent maternal anemia and enhance better oxygen delivery to the developing fetus [14]. Furthermore, studies have shown that FA supplementation reduces the risk of preterm labor, low birth weight, and gestational hypertension, thereby improving pregnancy outcomes [15]. Beyond its well-established roles in fetal development, FA also exhibits significant antioxidant and anti-inflammatory properties. It participates in various redox reactions as a coenzyme in the body [16,17]. FA, as an essential nutrient, plays a crucial role in supporting placental function and fetal development. Additionally, its potential to regulate oxidative stress makes it a compelling candidate for this study. In ruminants, it is generally accepted that the rumen microbiota synthesizes FA. However, Girard et al. concluded that lactating dairy cows with high FA requirements during pregnancy still require FA in their diets [18]. Therefore, FA supplementation during pregnancy is essential for both the mother and the fetus.

The nuclear factor erythroid-related factor 2 (NRF2) signaling pathway is an important mechanism for antioxidant stress in cells [19]. When cells are exposed to oxidants, the NRF2 signaling pathway is activated, which reduces the generation of ROS and enhances the activity of antioxidant enzymes [20,21]. Studies have shown that NRF2 signaling molecules can also influence the transcriptional activation of the mammalian target of rapamycin (mTOR), allowing cells to respond to changes in their external environment through the NRF2 and mTOR signaling pathways [22]. Adequate FA intake during pregnancy is essential for fetal and placental development, as FA deficiency has been linked to fetal growth restriction. In placental trophoblast cells of mice and humans, mTOR signaling can regulate fetal growth by controlling the transport of nutrients in the placenta [23,24]. Maternal folate deficiency has been reported to inhibit the placental mTOR signaling pathway, leading to fetal growth restriction [25]. Oxidative stress is caused by an imbalance between the production of ROS and the body’s antioxidant defense. When simulating oxidative stress in vitro, hydrogen peroxide (H_2_O_2_) is typically used as a stable and potent oxidant to induce ROS, which can effectively generate ROS in cells, leading to cell damage [26]. Oxidative stress induced by H_2_O_2_ can impair trophoblast cell function and disrupt placental homeostasis. However, it is unclear whether FA can alleviate H_2_O_2_-induced cellular oxidative damage by regulating the NRF2/mTOR signaling pathway when placental trophoblast cells are exposed to oxidative stress. Therefore, this study takes bovine placental trophoblast cells (BPTCs) as the research object, aiming to address whether FA can mitigate oxidative damage induced by H_2_O_2_ in BPTCs, focusing on its potential regulation of the NRF2/mTOR signaling pathway and to provide insight into its role in alleviating oxidative stress and improving placental cell function.

## 2. Results

### 2.1. Effect of Different Concentrations of H_2_O_2_ on the Cell Viability of BPTCs

**Figure 1:**
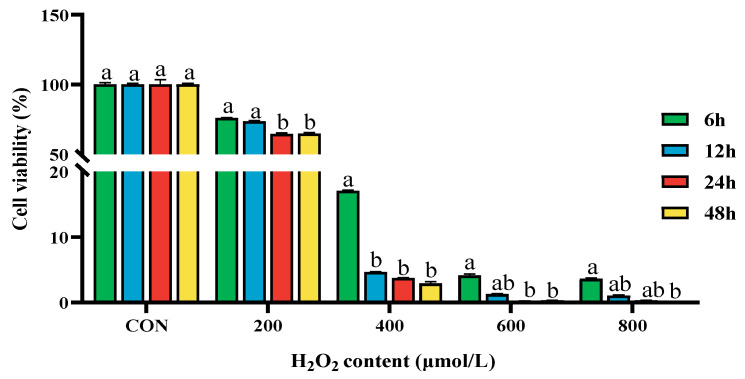
Effect of treating BPTCs with different concentrations of H_2_O_2_ (0, 200, 400, 600, and 800 μmol/L) for 6, 12, 24, and 48 h on cell viability. The results are presented as the mean (sample size, *n* = 6) and the standard error of the mean (SEM). Different superscripts (a–b) indicate significant differences.

When cells were treated with different concentrations of H_2_O_2_ for 24 h, the cells’ activity was significantly lower than that of the control group, and when the H_2_O_2_ concentration reached 200 μmol/L, the cell activity was significantly inhibited (*p* < 0.05, Figure 1).

### 2.2. Effect of Different Concentrations of H_2_O_2_ Treatment on the Cellular Content of ROS, MDA, LDH Activity, and Antioxidant Enzyme mRNA Expression After 24 h

**Figure 2:**
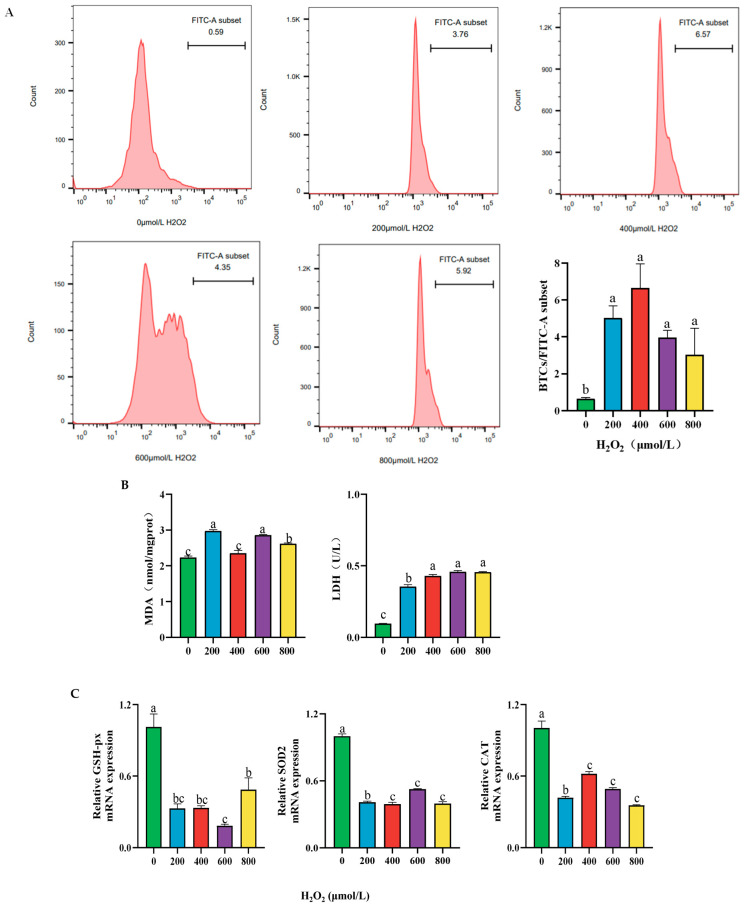
Effect of different concentrations of H_2_O_2_ treatment on cellular the content of ROS (**A**), LDH activity (**B**), MDA content (**B**), and *GSH-px* (**C**), *SOD2* (**C**), and *CAT* (**C**) gene expression after 24 h. The results are presented as the mean (sample size, *n* = 6) and the standard error of the mean (SEM). Different superscripts (a–c) indicate significant differences.

Compared with the control group, treatment with 200 μmol/L H_2_O_2_ for 24 h significantly increased the content of ROS, MDA in the cell, and LDH activity in the cell culture medium (*p* < 0.05; Figure 2A,B). Additionally, the mRNA expression of antioxidant enzyme genes *GSH-px*, *SOD2*, and *CAT* were significantly reduced in cells. (*p* < 0.05, Figure 2C).

### 2.3. Effect of Different Concentrations of H_2_O_2_ Treatment on Cell Apoptosis of BPTCs After 24 h

**Figure 3:**
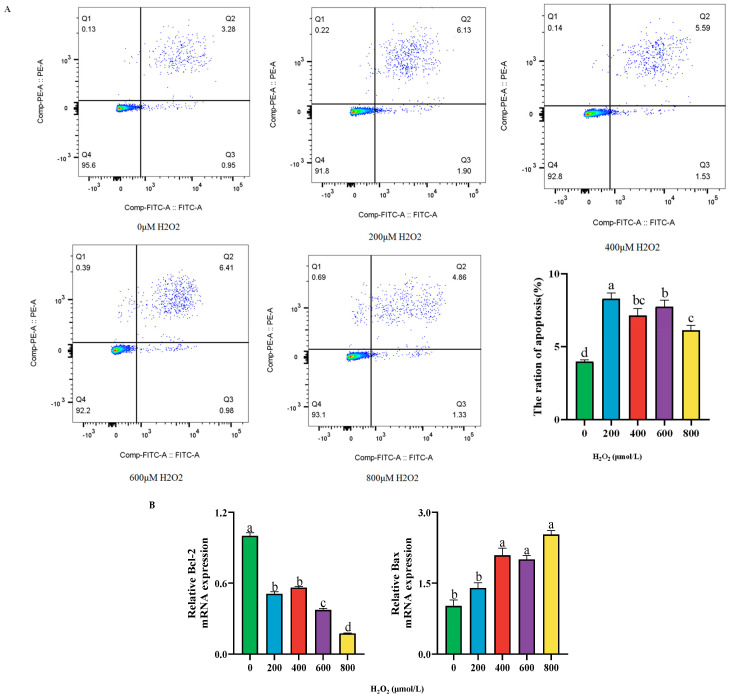
Effect of different concentrations of H_2_O_2_ treatment on the cell apoptosis rate (**A**) and the mRNA expression of apoptosis factor *Bcl-2* and *Bax* (**B**) after 24 h. The results are presented as the mean (sample size, *n* = 6) and the standard error of the mean (SEM). Different superscripts (a–d) indicate significant differences.

Compared to the control group, the apoptosis rate of cells significantly increased after 24 h of treatment with 200 μmol/L H_2_O_2_ (*p* < 0.05, Figure 3A). In addition, it was found that after H_2_O_2_ treatment, the mRNA expression of the anti-inflammatory factor *Bcl-2* was significantly lower than that in the control group (*p* < 0.05, Figure 3B), while the mRNA expression of the pro-inflammatory factor *Bax* was significantly higher (*p* < 0.05, Figure 3B). Therefore, this study determined to establish a cellular oxidative stress model by treating cells with 200 μmol/L H_2_O_2_ for 24 h.

### 2.4. Effect of Different Concentrations of FA and H_2_O_2_ on the Cell Viability of BPTCs

**Figure 4:**
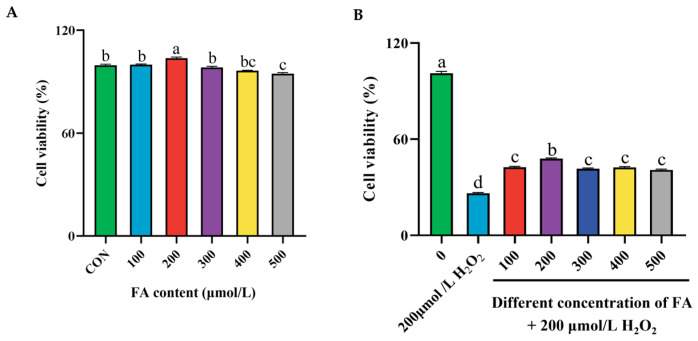
Effect of different concentrations of FA (**A**) and FA+ H_2_O_2_ (**B**) on the cell viability of BPTCs. The results are presented as the mean (sample size, *n* = 6) and the standard error of the mean (SEM). Different superscripts (a–d) indicate significant differences.

According to the results of the CCK-8 assay, compared with the control group, when the FA concentration reached 200 μmol/L, the cell viability significantly increased after 12 h of treatment with different concentrations of FA (*p* < 0.05, Figure 4A). In contrast, when cells were pretreated with different concentrations of FA for 12 h and then cotreated with 200 μmol/L H_2_O_2_ for 24 h, the cell viability of the group treated with 200 μmol/L FA and H_2_O_2_ was significantly higher than that of the cells treated with 200 μmol/L H_2_O_2_ alone (*p* < 0.05, Figure 4B). Therefore, this study decided to establish a cellular oxidative stress relief model for subsequent experiments by pretreating cells with 200 μmol/L FA for 12 h and then cotreating them with 200 μmol/L H_2_O_2_ for 24 h.

### 2.5. Effect of FA on H_2_O_2_-Induced Oxidative Stress in BPTCs

#### 2.5.1. Effect of FA on ROS, MDA, and T-AOC Content, LDH and SOD Activity, and Antioxidant Enzyme Gene Expression Induced by H_2_O_2_ in BPTCs

**Figure 5:**
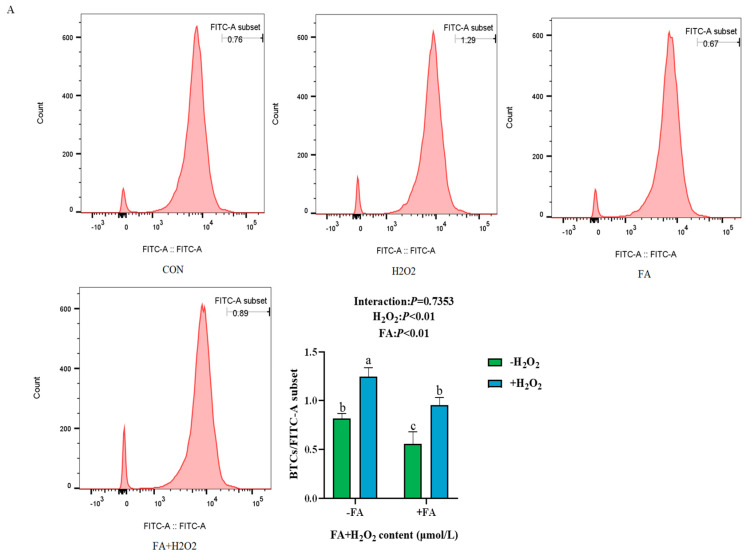

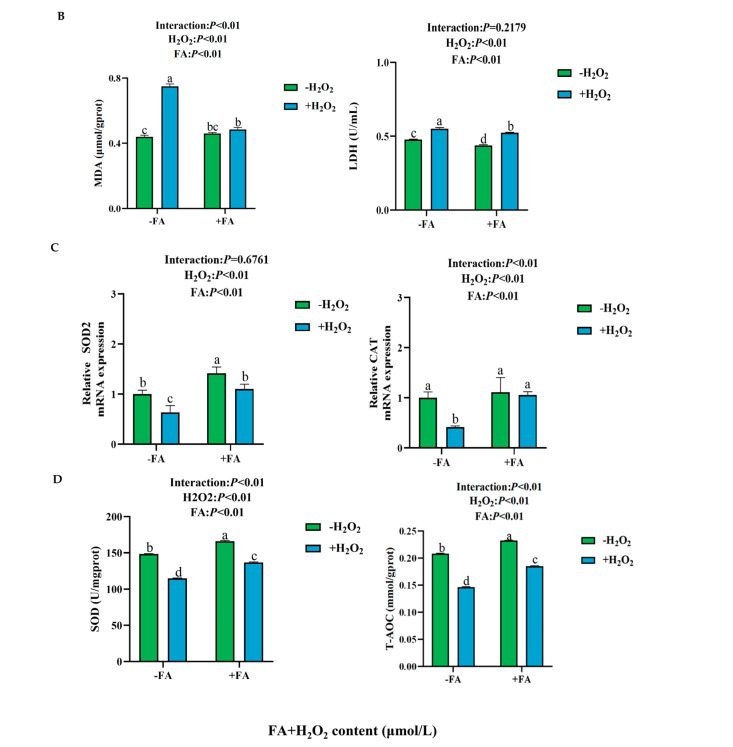
Effect of FA on H_2_O_2_-induced ROS (**A**), MDA content (**B**), and LDH activity (**B**), antioxidant enzyme *SOD2* and *CAT* mRNA expression (**C**), and antioxidant enzyme activity SOD and T-AOC content (**D**) in BPTCs. The results are presented as the mean (sample size, *n* = 6) and the standard error of the mean (SEM). Different superscripts (a–d) indicate significant differences.

Compared to the control group, the H_2_O_2_ group exhibited a significant increase in the content of ROS, MDA, and LDH activity (Figure 5A,B; *p* < 0.05). However, these were significantly reduced in the FA+ H_2_O_2_ group compared to the H_2_O_2_ group (*p* < 0.05; Figure 5A,B). As shown in Figure 5C, the mRNA expression levels of *SOD2* and *CAT* in both the control group and the FA+ H_2_O_2_ group were significantly higher than those in the H_2_O_2_ group (*p* < 0.05). Additionally, the results of the intracellular antioxidant enzyme activity (SOD) and T-AOC content detection showed that, compared with the control group, the H_2_O_2_ group significantly decreased SOD enzyme activity and T-AOC content in the cells, whereas FA treatment significantly increased their levels (*p* < 0.05, Figure 5D).

#### 2.5.2. Effect of FA on H_2_O_2_-Induced Apoptosis and Cell Proliferation of BPTCs

**Figure 6:**
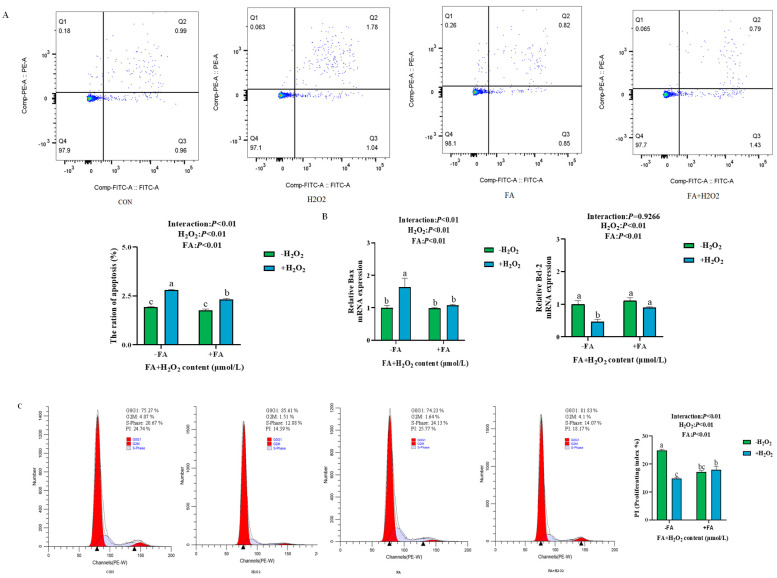
Effect of FA on H_2_O_2_-induced cell apoptosis (**A**), the mRNA expression of apoptosis factor *Bax* and *Bcl-2* (**B**), and cell proliferation (**C**) of BPTCs (▲: indicating the key positions of different cell cycle stages in the distribution of DNA content). The results are presented as the mean (sample size, *n* = 6) and the standard error of the mean (SEM). Different superscripts (a–c) indicate significant differences.

Compared to the control group, the apoptosis rate of cells was significantly increased in the H_2_O_2_ group (*p* < 0.05, Figure 6A). However, the apoptosis rate of cells in the FA+ H_2_O_2_ group was significantly reduced in comparison to the H_2_O_2_ group (*p* < 0.05, Figure 6A). In addition, the expression of apoptosis-related genes was assessed, revealing that the relative mRNA expression of the anti-inflammatory factor *Bcl-2* in the H_2_O_2_ group was significantly lower than that in the control group (*p* < 0.05, Figure 6B), while the mRNA expression of the pro-inflammatory factor *Bax* was significantly higher (*p* < 0.05, Figure 6B). As shown in Figure 6C, the S phase and propidium iodide (PI) values of trophoblast cells were significantly reduced after H_2_O_2_ treatment compared with the control group (*p* < 0.05), whereas the PI values of cells in the FA+ H_2_O_2_ group increased (*p* < 0.05) compared to the H_2_O_2_ group.

### 2.6. Effects of FA on H_2_O_2_ Induced the Damage of Tight Junctions, Nutrient Transporters, and Cell Functional Factors in BPTCs

**Figure 7:**
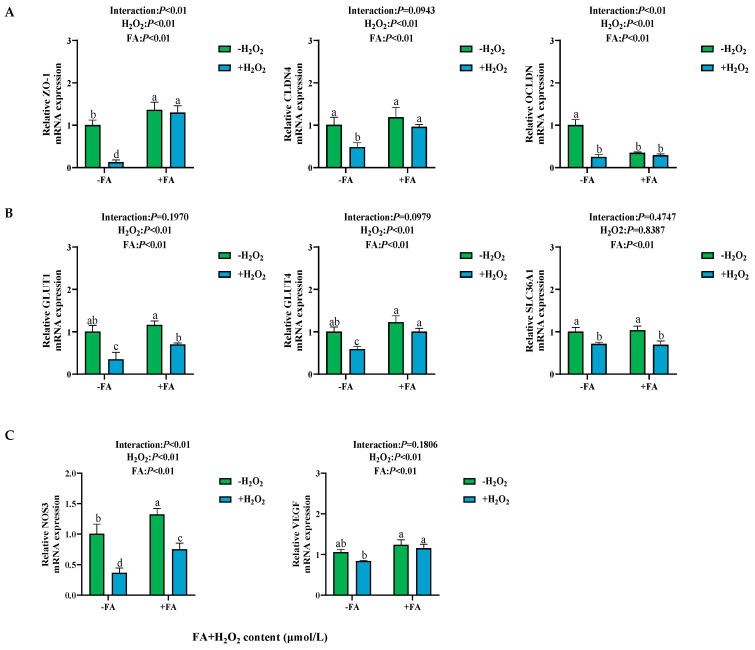
Effect of FA on H_2_O_2_-induced tight junctions (**A**) and nutrient transporters (**B**) and functional factors (**C**) in BPTCs. The results are presented as the mean (sample size, *n* = 6) and the standard error of the mean (SEM). Different superscripts (a–d) indicate significant differences.

The treatment with H_2_O_2_ resulted in a significant reduction in the relative mRNA expression levels of *ZO-1* and *CLDN4* (*p* < 0.05, Figure 7A). Conversely, in the FA+ H_2_O_2_ group, the relative mRNA expression levels of *ZO-1* and *CLDN4* were increased compared to the H_2_O_2_ group (*p* < 0.05, Figure 7B). In addition, it was observed that the relative mRNA expression levels of *GLUT1*, *GLUT4*, and *SLC36A1* (solute carrier family 36 member 1) in the H_2_O_2_ group were decreased compared to the control group (*p* < 0.05, Figure 7B). Furthermore, the relative mRNA expression levels of *NOS3* and *VEGF* of BPTCs in the FA+ H_2_O_2_ group increased compared to the H_2_O_2_ group (*p* < 0.05, Figure 7C).

### 2.7. Effect of FA on the Expression of NRF2/mTOR Signaling Pathway-Related Genes in BPTCs Induced by H_2_O_2_

**Figure 8:**
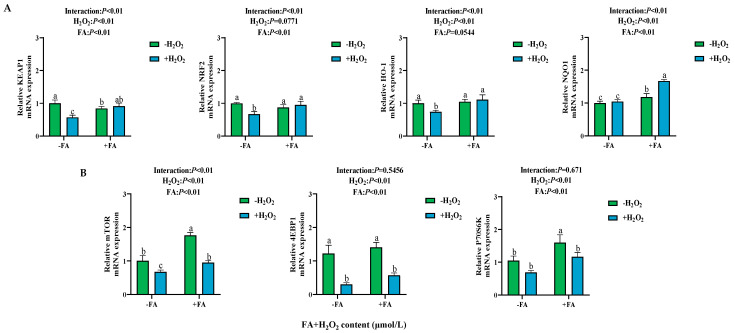
Effect of FA on the expression of NRF2/mTOR signaling pathway-related genes in BPTCs induced by H_2_O_2_ (**A**,**B**). The results are presented as the mean (sample size, *n* = 6) and the standard error of the mean (SEM). Different superscripts (a–c) indicate significant differences.

H_2_O_2_ treatment reduced the mRNA expression of *KEAP1*, *NRF2*, *HO-1*, *mTOR*, *P70S6K*, and *4EBP1* (*p* < 0.05; Figure 8A,B). After adding FA pretreatment, the FA+ H_2_O_2_ group showed an increase in *KEAP1*, *NRF2*, *HO-1*, *NQO1*, *mTOR*, *P70S6K*, and *4EBP1* mRNA expression (*p* < 0.05; Figure 8A,B).

### 2.8. Effect of FA on the Abundance of NRF2/mTOR Signaling Pathway-Related Proteins in BPTCs Induced by H_2_O_2_

**Figure 9:**
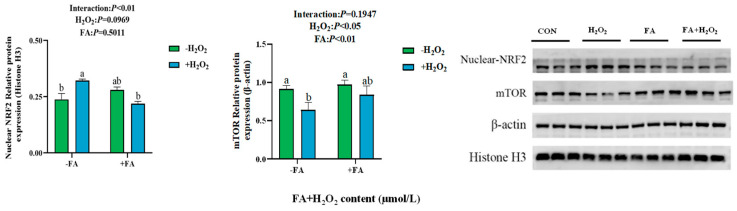
Effect of FA on the abundance of NRF2/mTOR signaling pathway-related proteins in BPTCs induced by H_2_O_2_. The results are presented as the mean (sample size, *n* = 3) and the standard error of the mean (SEM). Different superscripts (a–b) indicate significant differences.

The protein abundance results showed that, compared to the control group, the protein abundance of mTOR in the H_2_O_2_ group was significantly decreased, while the levels of NRF2 in the nucleus of the H_2_O_2_ group were significantly higher than those in the control group (*p* < 0.05, Figure 9). After adding FA pretreatment, the FA+ H_2_O_2_ group exhibited a decrease in the protein abundance of nuclear NRF2 and an increasing trend in mTOR protein abundance (Figure 9).

### 2.9. Effect of ML385 (NRF2 Inhibitor) on FA-Mediated NRF2/mTOR Signaling Pathway in BPTCs

#### 2.9.1. Effect of Different Concentrations of ML385 and Combined Treatment with FA, H_2_O_2_, and ML385 on Cell Viability

**Figure 10:**
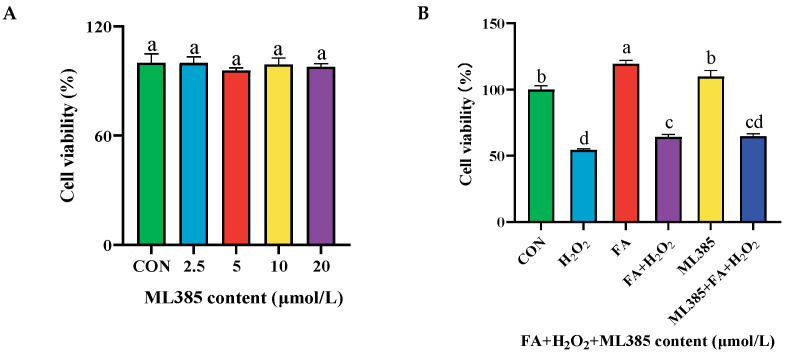
Effect of different concentrations of ML385 (**A**) and combined treatment with FA, H_2_O_2_, and ML385 (**B**) on cell viability. The results are presented as the mean (sample size, *n* = 6) and the standard error of the mean (SEM). Different superscripts (a–d) indicate significant differences.

To research the mechanism of FA alleviating H_2_O_2_-induced oxidative stress in trophoblast cells, the NRF2 inhibitor ML385 was used. The different concentrations of ML385 (2.5, 5, 10, 20 μmol/L) had no significant effect on the cell viability of BPTCs compared with the control group (Figure 10A). Therefore, 20 μmol/L ML385 was chosen for subsequent tests. When ML385 interacts with the FA+ H_2_O_2_ group, there is no significant difference compared to the H_2_O_2_ group, indicating that ML385 does not affect the effect of FA and H_2_O_2_ on BPTCs (*p* > 0.05, Figure 10B).

#### 2.9.2. Effect of Combined Treatment with FA, H_2_O_2_, and ML385 on the Gene Expressions of Antioxidant Enzyme in BPTCs

**Figure 11:**
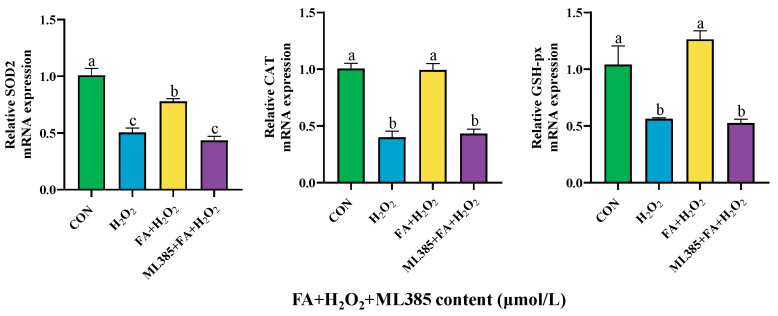
Effect of ML385 (NRF2 inhibitor) on the expression of antioxidant enzyme genes in BPTC. The results are presented as the mean (sample size, *n* = 6) and the standard error of the mean (SEM). Different superscripts (a–c) indicate significant differences.

The results showed that, compared to the control group, the mRNA expression levels of *SOD2*, *CAT*, and *GSH-px* in BPTCs in the H_2_O_2_ group were lower (*p* < 0.05, Figure 10). Compared with the H_2_O_2_ group, the mRNA expression levels of *SOD2*, *CAT*, and *GSH-px* in BPTCs were significantly increased in the FA+ H_2_O_2_ group (*p* < 0.05, Figure 11). However, compared with the FA+ H_2_O_2_ group, the mRNA expression levels of antioxidant enzyme genes in the ML385+ FA+ H_2_O_2_ group were significantly reduced (*p* < 0.05, Figure 11).

#### 2.9.3. Effect of Combined Treatment with FA, H_2_O_2_, and ML385 on the mRNA Expression of Apoptosis Factor in BPTCs

**Figure 12:**
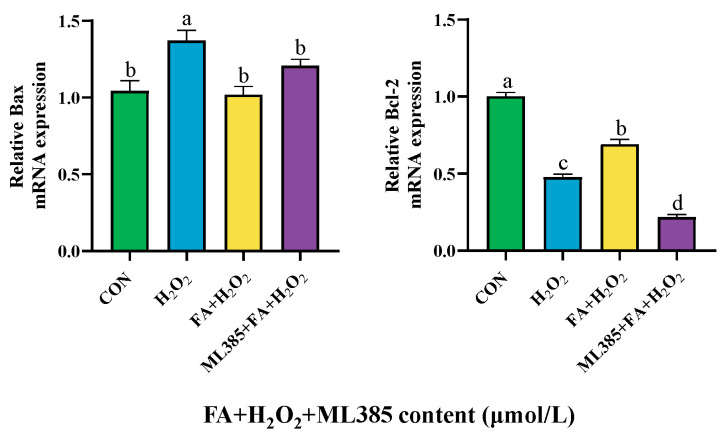
Effect of ML385 (NRF2 inhibitor) on the expression of apoptotic factors of BPTCs. The results are presented as the mean (sample size, *n* = 6) and the standard error of the mean (SEM). Different superscripts (a–d) indicate significant differences.

Further detection of the relative mRNA expression of *Bax* in cells in the ML385+ FA+ H_2_O_2_ group increased, while reducing the relative mRNA expression of *Bcl-2* (Figure 12).

#### 2.9.4. Effect of Combined Treatment with FA, H_2_O_2_, and ML385 on the mRNA Expression of Tight Junctions, Nutrient Transporters, and Cell Functional Factors in BPTCs

**Figure 13:**
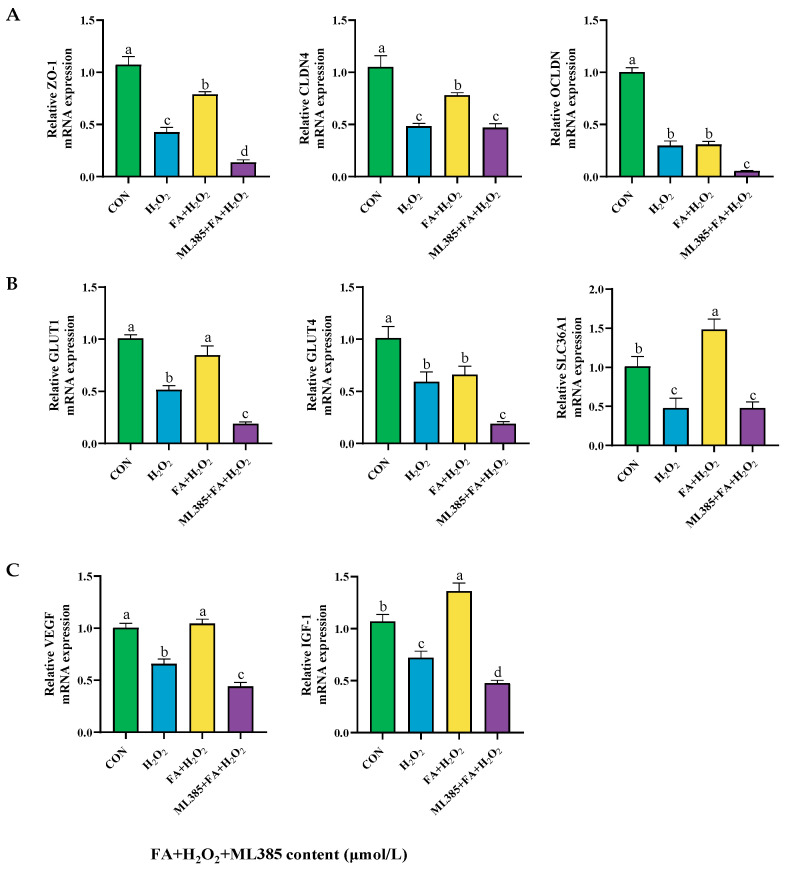
Effect of ML385 (NRF2 inhibitor) on the mRNA expression of tight junction factors (**A**), nutrient transporters (**B**), and functional factors (**C**) in BPTCs. The results are presented as the mean (sample size, *n* = 6) and the standard error of the mean (SEM). Different superscripts (a–d) indicate significant differences.

Compared with the H*_2_*O*_2_* group, the relative mRNA expression levels of tight junction-related genes *ZO-1*, *CLDN4*, and *OCLDN* were significantly decreased (*p* < 0.05, Figure 13A). Similarly, the expression levels of nutrient transporter genes *GLUT1*, *GLUT4*, and *SLC36A1* were also reduced (*p* < 0.05, Figure 13B). Additionally, the expression of trophoblast cytokine genes *VEGF* and *IGF-1* was significantly reduced in the ML385+ FA+ H*_2_*O*_2_* group (*p* < 0.05, Figure 13C).

### 2.10. Effect of Combined Treatment with FA, H_2_O_2_, and ML385 on the Expression of Cellular Pathway Genes

**Figure 14:**
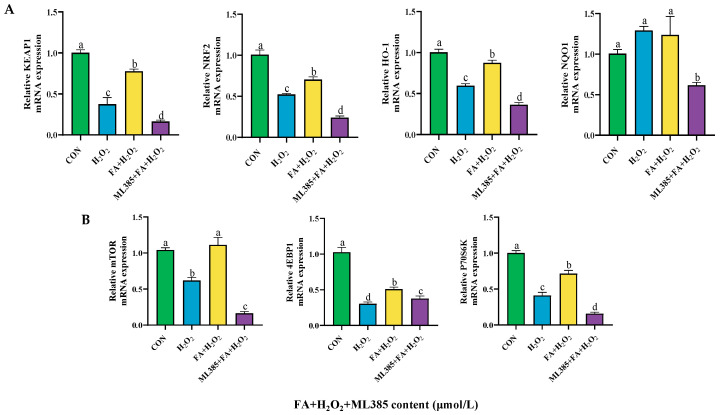
Effect of ML385 on the gene expression of FA-mediated NRF2/mTOR signaling pathway in BPTCs (**A**,**B**). The results are presented as the mean (sample size, *n* = 6) and the standard error of the mean (SEM). Different superscripts (a–d) indicate significant differences.

In addition, compared with the FA+ H_2_O_2_ group, the ML385+FA+ H_2_O_2_ group significantly reduced the mRNA expression of *KEAP1*, *NRF2*, *HO-1*, *NQO1*, *mTOR*, *4EBP1*, and *P70S6K* (*p* < 0.05; Figure 14A,B).

### 2.11. Effect of Combined Treatment with FA, H_2_O_2_, and ML385 on the Abundance of Cellular Pathway Proteins

**Figure 15:**
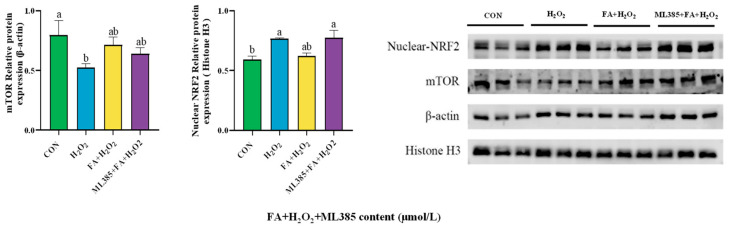
Effect of ML385 on FA-mediated NRF2/mTOR signaling pathway in BPTCs. The results are presented as the mean (sample size, *n* = 3) and the standard error of the mean (SEM). Different superscripts (a–b) indicate significant differences.

The protein abundance of mTOR in cells showed a decreasing trend, while the NRF2 in the nucleus was increased (*p* < 0.05, Figure 15).

## 3. Discussion

When the rate of ROS production in living cells exceeds the rate at which they are utilized, the body accumulates excessive ROS, leading to oxidative stress [27]. This phenomenon occurs during pregnancy in cows due to a negative energy balance during the transition period, heat stress, and other external environmental factors [28,29,30]. Studies indicate that excessive ROS production results in oxidative stress in the mother, which can damage the placenta [31,32]. Therefore, utilizing BPTCs as a research model to investigate the effects of adding antioxidants to alleviate oxidative stress in pregnant cows and their fetuses is of great significance for enhancing cow production efficiency. However, in vitro models cannot fully simulate the environmental changes that occur in vivo. When oxidative stress occurs in an animal, the intensity of cellular peroxidative damage and antioxidant defenses changes dynamically due to the complexity of the in vivo environment. The organism’s own antioxidant defenses are likely to be activated, and the levels of antioxidant factors in the body may increase in direct response to this adverse stress.

The occurrence of retained placenta (RP) in bovines is closely associated with increased oxidative stress within placental tissues. The activities of key antioxidant enzymes (e.g., GSH-px, glutathione transferase (GST), CAT, and SOD) are significantly reduced in retained placenta compared to normally detached placenta, making it more susceptible to oxidative damage [33]. Additionally, a compromised antioxidant defense system may disrupt the normal functioning of placental tissues, leading to cellular damage and dysfunction. Studies conducted on human placental trophoblast cells HTR-8/SVneo have revealed that maternal preeclampsia is linked to maternal oxidative stress. When HTR-8/Svneo cells were treated with 200 μmol/L H_2_O_2_, both the cell viability and content of antioxidants such as GSH-px, SOD, and CAT were significantly reduced. On the other hand, the content of intracellular ROS and MDA was increased [34]. These findings suggest that the cell viability of placental trophoblast cells is negatively affected in the presence of H_2_O_2_. Furthermore, in this study, the content of intracellular ROS, MDA, and LDH activity was found to be significantly higher in the H_2_O_2_ group than the control group, while the gene expression of antioxidant enzymes genes like *SOD2*, *GSH-px*, SOD enzyme activity, and T-AOC were lower compared with the control group. Oxidative stress can trigger additional pathways in cells, such as cellular apoptosis and immune response [35]. Li et al. found in their study on human first-trimester trophoblast/simian virus (HTR8/SVneo) cells that the apoptosis rate of placental trophoblast cells increases with the increase in H_2_O_2_ concentration, and the content of intracellular antioxidant enzymes decreases [36]. In early pregnancy stages, placental trophoblast cells are often in a hypoxic oxidative stress state, which leads to an increase in the cell apoptosis rate and reduced proliferation [37]. This study also revealed that the detection of LDH activity in the culture medium increased, and the structure of the cell membrane was disrupted. The apoptosis rate of cells and the activity of LDH were significantly increased, and the mRNA expression of intracellular pro-inflammatory factor *Bax* was higher, while the expression of anti-inflammatory factor *Bcl-2* was lower.

VEGF, NOS3, and IGF-1 are essential for normal placental function and angiogenesis [38]. Oxidative stress impairs placental angiogenesis, which is a critical indicator of the placental barrier phenotype [39]. FA treatment has been shown to alleviate oxidative damage in human trophoblast cells induced by tert-butyl hydroperoxide (TBHP) [40]. In the present study, we observed that the presence of H_2_O_2_ led to a decrease in the expression of trophoblast functional factors and angiogenic factors, while FA pretreatment alleviated this effect caused by H_2_O_2_. The mammalian placenta consists of trophoblast cells that form a tight barrier to protect the fetus [41]. Two proteins, ZO-1 and E-cadherin, are highly expressed in the mammalian placenta and are crucial components of the placental barrier [8,32]. Li et al. reported that H_2_O_2_ induced oxidative stress in human trophoblast cells, resulting in a decreased abundance of ZO-1 and Occludin proteins, which impaired cell invasion and tubular formation [36]. Huang et al. found that the Occludin/ZO-1 signaling pathway may be involved in protecting the placental barrier from oxidative stress and counteracting the effects of antioxidants on trophoblast cells when porcine placental trophoblast cells were used in vitro to simulate placental oxidative stress [42]. The mammalian placental barrier primarily consists of a single trophoblast layer and an endothelial vascular barrier that selectively transports nutrients and toxins [43]. Previous studies have shown that disruption of the placental barrier may allow pathogens or harmful metabolites to affect placental and fetal tissues [44]. Therefore, we hypothesized that H_2_O_2_ transit through the placenta may impair placental barrier function. In the current study, we found that the effects of H_2_O_2_ on *ZO-1* and *Claudin-4* expression in placental trophoblast cells were attenuated by the antioxidant folic acid; however, this study did not explore whether FA could attenuate the effects of oxidative stress on placental barrier permeability. In addition, we examined the effects of oxidative stress on placental nutrient transporters following impaired placental barrier function in BPTCs. The results revealed that the mRNA expression of major nutrient transport proteins, such as *GLUT1*, *GLUT4*, and *SLC36A1*, was downregulated in the H_2_O_2_ group. However, FA pretreatment led to the upregulation of the mRNA expression of all these transporters. Therefore, we suggest that antioxidants have a mitigating effect on oxidation-induced impairment of the placental barrier nutrient transport function. However, further studies are needed to investigate the underlying mechanisms of placental barrier disruption and the adverse pregnancy outcomes caused by maternal oxidative stress.

Numerous studies have demonstrated that the mTOR signaling pathway plays a crucial central role in placental trophoblast cells [45]. In the placenta, mTOR responds to various growth-related signals, including amino acids, glucose, oxygen, folate, and growth factors. It regulates nutrient transport and protein synthesis in placental trophoblast cells, which in turn influences fetal growth [25,46]. Furthermore, Bendavit et al. have discovered that the NRF2 transcription factor can directly modulate the activity of mTOR [22]. In this experiment, it was observed that after oxidative stress was induced in bovine placental trophoblast cells (BPTCs), the relative mRNA expression of the mTOR signaling pathway, as well as the NRF2 signaling pathway and its downstream-related antioxidant signaling molecules in the cells, decreased. However, we found that the protein abundance of mTOR decreased, while the expression of NRF2 in the nucleus increased, indicating that after inducing oxidative stress in trophoblast cells with H_2_O_2_, the NRF2 and mTOR signaling pathways were activated. It has been established that, under normal conditions, KEAP1 binds to NRF2 in the cytoplasm. However, under stress conditions, KEAP1 becomes inactive, NRF2 is released from KEAP1, and NRF2 undergoes nuclear translocation [47]. Zou et al. found that after H_2_O_2_ treatment of HepG2 cells, the mRNA expression of antioxidant factors *HO-1* and *NQO1* in the cells decreased, which is consistent with the results of this experiment [48]. According to Wei et al., when pig intestinal epithelial cells undergo oxidative stress, the abundance of NRF2 protein in the nucleus increases [49]. To investigate the mechanism by which FA alleviates oxidative damage in bovine placental trophoblast cells, we examined the effect of NRF2 pathway inhibitors on the mechanism of FA relief. Our research revealed that the NRF2 pathway inhibitor ML385 reversed the protective effect of FA against cellular oxidative damage. There is evidence to suggest that ML385 is a novel inhibitor that blocks NRF2 and regulates the expression of its downstream target genes. Studies have shown that when prooxidants are used to induce oxidative stress in mice, cotreatment with ML385 exacerbates oxidative stress [50,51]. Meanwhile, Song et al. found that both the use of the NRF2 pathway inhibitor ML385 and the knockout of NRF2 can significantly reverse the antioxidant’s alleviating effect on antioxidants’ oxidative stress [52].

These experimental data indicate that the study of inhibitors has validated changes in the abundance of signaling pathway proteins through techniques such as Western blotting. This confirms that FA alleviates oxidative stress in bovine placental trophoblast cells by regulating the NRF2/mTOR signaling pathway. In this study, we found that FA pretreatment significantly increased the activity of the antioxidant enzyme SOD and the content of T-AOC in the cells. However, after treatment with ML385, we only detected the expression levels of genes related to antioxidant enzymes; we did not measure enzyme activity. Therefore, further research is needed to investigate changes in antioxidant enzyme activity and enzyme spatial structure, as well as to clarify the mechanism by which folate regulates oxidative stress in trophoblast cells.

## 4. Materials and Methods

### 4.1. Cell Culture and Treatment

The BPTCs were provided by the Beijing Academy of Agricultural Sciences (Beijing, China) [53]. The general steps for establishing immortalized cell lines are as follows: placental tissue was taken from the placenta of a cow that was 45–60 d pregnant, cultured to form monolayer cells, transfected with pC1-neo-hTERT eukaryotic expression vector, screened with G418, and cultured to 50 generations, then immortalized bovine placental trophoblast cell lines were then obtained. The cell lines used in this experiment were 50 generations, and the cells were passed 2–3 times during the experiment. Cell identification tests were also performed under culture conditions in our laboratory [54]. The cultivation conditions are as follows: the cells were cultured in DMEM/F-12 medium (Gibco, Grand Island, NE, USA) supplemented with 10% fetal bovine serum (Gibco, Grand Island, NE, USA) and 1% triple antibiotic (10 kU/mL penicillin, 10 mg/mL streptomycin, 25 µg/mL amphotericin B, SolelyBio, Beijing, China) at 37 °C and 5% CO_2_ in an incubator (Thermo Fisher, Waltham, MA, USA). Various concentrations of H_2_O_2_ (0, 200, 400, 600, and 800 μmol/L) (Sigma Chemical Co., St. Louis, MO, USA) were used to treat cells for 6, 12, 24, and 48 h, respectively, and then suitable concentrations and treatment times were selected to establish a cell oxidative damage model. Additionally, cells were pretreated with FA (0, 100, 200, 300, 400, and 500 μmol/L) (Sigma Chemical Co., St. Louis, MO, USA) for 12 h before exposure to H_2_O_2_ to establish a model for mitigating cellular oxidative damage.

### 4.2. Cell Viability Assay

According to the manufacturer’s instructions, cell viability was assessed using the CCK-8 (Cell Counting Kit-8, Auresis, Chengdu, China). A total of 2 × 10³ cells/mL were inoculated into 96-well plates, with the blank treatment group receiving cell-free complete culture medium. Once the cells reached 80% confluence, the old culture medium was discarded, and the prepared treatment medium was added. The cells were then treated as described previously for cell culture and treatment. Subsequently, 10 μL of CCK-8 solution was added to each well, and the plates were incubated at 37 °C in a 5% CO_2_ incubator for 1 h. Finally, the absorbance values at 450 nm were measured using a microplate reader (Bio-Rad, Hercules, CA, USA).

### 4.3. Flow Cytometric Analysis of Cell Cycle

According to the manufacturer’s instructions, the cell cycle was analyzed using flow cytometry and the propidium iodide (PI)/RNase detection kit (BD Biosciences, Franklin Lakes, NJ, USA). A total of 1 × 10^5^ cells/mL were inoculated into 6-well plates and cultured until they reached 80% confluence. The old medium was discarded and the cells treated with the treatment medium prepared as described earlier. Briefly, add 1 mL of pre-cooled 70% ethanol to fix the cells overnight at 4 °C. Centrifuge the cells at 1000× *g* for 5 min and wash them twice. Stain the cells with 0.5 mL of PI solution and incubate them in the dark at room temperature for 30 min. Detect fluorescence at an excitation wavelength of 488 nm and an emission wavelength of 585 ± 21 nm using flow cytometry, and calculate the cell proliferation index (PI) with the formula PI = (S + G_2_/M)/(G_0_/G_1_ + S + G_2_/M) [35,55]. Analyze the data statistically using ModFit LT 5.0 software.

### 4.4. ROS Assay

The DCFH-DA probe dye kit (Solaibao, Beijing, China) was used to detect the ROS content in cells. The BPTCs were inoculated with 1 × 10^5^ cells/mL in 12-well plates. After treatment as described earlier, cells were resuspended in DCFH-DA probe dye diluted with serum-free culture medium at 1:1000, incubated for 20 min at 37 °C, and washed three times with serum-free cell culture medium. Finally, the fluorescence intensity of cells at the 488 nm excitation wavelength and 525 nm emission wavelength were detected by flow cytometry.

### 4.5. MDA, T-AOC Content, LDH, and SOD Activity Assays

The following procedures were performed according to the instructions provided with the Malondialdehyde (MDA), Lactate Dehydrogenase (LDH), Superoxide Dismutase (SOD), and Total Antioxidant Capacity (T-AOC) Assay Kits (Nanjing Built, Nanjing, China). MDA is a marker of lipid peroxidation, T-AOC reflects the total antioxidant capacity of cells, SOD is an important antioxidant enzyme that scavenges superoxide radicals, and LDH is an indicator of cell membrane integrity and cytotoxicity. After collecting the cells, the total protein concentration was determined using a BCA protein concentration assay kit (Abbkine, Wuhan, China). The cells were lysed using an ultrasonic cell pulverizer (Servicebio, Wuhan, China). The absorbance of MDA at 530 nm, LDH and SOD at 450 nm, and T-AOC at 425 nm in the cell samples was measured using a fluorescent enzyme marker (BioTeK, Winooski, VT, USA).

### 4.6. Cell Apoptosis Assay

The fluorescein isothiocyanate (FITC) annexin V apoptosis detection kit (DOJINDO, Kyushu Island, Japan) was used to analyze cell apoptosis by flow cytometry. First, 1 × 10^5^ cells/mL were inoculated in 12-well plates, and the medium and precipitate of cells were collected after treatment. The cells were then washed twice in PBS, following which the prepared 1 ×Annexin V Binding Solution was added to them. A new tube was used to add 100 μL of the prepared cell suspension, followed by 5 μL of Annexin V, FITC, and 5 μL of PI Solution. The tubes were then incubated at room temperature for 15 min in the dark, and 400 μL of 1 ×Annexin V Binding Solution was added to each tube. Finally, all the samples were analyzed through a flow cytometer at the 488 nm excitation wavelength and 530 nm emission wavelength.

### 4.7. RNA Extraction and Quantitative Real-Time PCR

The Steady Pure Universal RNA Extraction Kit (Accurate Biotechnology, Hunan, China) was used to extract and purify total RNA from BPTCs following treatment. The RNA concentration was measured using a NanoDrop ND 2000 spectrophotometer (Thermo Scientific, Waltham, MA, USA), and RNA quality was assessed by calculating the ratio of UV absorption at 260 nm and 280 nm (A260/A280). RNA samples were diluted to a concentration of 30 ng/μL. The Toyobo Reverse Transcription Kit (ReverTra Ace^®^ QPCR RT Master Mix, Servicebio, Wuhan, China) was used to reverse transcribe RNA into cDNA. Real-time fluorescent quantitative PCR (qPCR) was performed using the Fast Fire qPCR PreMix (SYBR Green) kit (Vazyme, Nanjing, China). The results were normalized to the expression of β-actin and calculated using the 2^−ΔΔCT^ method [56]. The primer sequences used in this study are listed in Table 1 (Sangon Biotech, Shanghai, China).

### 4.8. Western Blot

Total cell proteins were extracted using the SevenFast^®^ The Column Total Protein Extraction Kit (Abbkine, Wuhan, China). Nuclear proteins were extracted according to the instructions of the reagent kit (Solebao, Beijing, China). The concentrations of total protein and nuclear protein in the cells were determined using the BCA protein concentration assay kit (Biosharp, Guangzhou, China). Briefly, protein analysis was performed as follows: A 7.5% PAGE gel was prepared using a rapid preparation kit (Epizyme, Shanghai, China) to isolate the proteins, which were then transferred to a PVDF membrane (Bio-Rad, Hercules, CA, USA). The membrane was blocked with a rapid-blocking solution (Saville, Wuhan, China) and incubated overnight at 4 °C with primary antibodies: NRF2 (1:2000, rabbit-derived, ABclonal, Wuhan, China), mTOR (1:500, rabbit-derived, Bioss, Beijing, China), β-actin (1:500, rabbit-derived, Bioss, Beijing, China) and Histone H3 (1:500, rabbit-derived, ABclonal, Wuhan, China). The membrane was then incubated with goat anti-rabbit IgG (1:5000, ABclonal, Wuhan, China) secondary antibody conjugated with horseradish peroxidase (HRP) at room temperature. Subsequently, the membrane was incubated with a specific ultra-sensitive ECL chemiluminescent substrate (Oriscience, Chengdu, China), and the protein bands were analyzed using a ChemiDoc™ Imaging System (Bio-Rad, CA, USA). Grayscale analysis was performed using ImageJ 1.8.0 software.

### 4.9. Statistical Analysis

In this study, all data were analyzed for one-way ANOVA using the general linear model of SPSS statistical software 27.0.1, and multiple comparisons were conducted using the Tukey method. All data were represented as the mean and standard error, with *p* < 0.05 indicating significant differences.

## 5. Conclusions

In conclusion, this study confirmed that FA alleviates H_2_O_2_-induced oxidative damage in bovine placental trophoblast cells by regulating the NRF2/mTOR signaling pathway. FA enhances cell proliferation, increases the activity of antioxidant enzymes and gene expression, reduces cell apoptosis caused by oxidative damage, and improves the placental barrier function composed of trophoblasts. These findings suggest that FA supplementation could be a promising strategy to alleviate oxidative stress caused by physiological, pathological, and environmental factors in cows during pregnancy. However, in vitro cell studies cannot fully represent the complex mechanisms of oxidative stress during pregnancy in cows. Therefore, it is necessary to conduct in vivo experiments to further explore the regulatory effects of the optimal dose and timing of FA supplementation on oxidative stress throughout the pregnancy of cows. This research will also help clarify how the absorption and metabolism of FA influence the structure and function of the placenta, thereby affecting fetal growth and development, which is of great significance and value for translating these findings into practical applications in pregnant cows.

## Figures and Tables

**Table 1:** Primer sequences for quantitative real-time PCR This is a table.

**Gene** **s**	**Forward (5′** **→** **3** **′** **)**	**Reverse (5′** **→** **3′)**	**Accession No.**
*CAT*	TCACTCAGGTGCGGACTTTC	TCTCACACAGGCGTTTCCTC	NM_001035386.2
*SOD2*	GGGTTGGCTCGGCTTCAATAAGG	TCGTGCAGTTACATTCTCCCAGTTG	NM_201527.2
*GSH-px*	TCGCAATGAGGCAAGACTGACG	TAGCACGGCAGGTCCTTCTCTATC	NM_001346430.1
*Bcl-2*	ATGTGTGTGGAGAGCGTCAA	GTGCCTTCAGAGACAGCCAG	NM_001166486.1
*Bax*	TGCTTCAGGGTTTCATCCAG GG	GTCCTGATCAACTCGGGCAC	NM_173894.1
*GLUT1*	TGGGCTTCTCAAAACTGGG	GGATGCCGACGACGATG	NM_174602.2
*GLUT4*	AGGAGGAGAAGCGGAAGC	AATGGCGATGACGAGGG	NM_174604.1
*SLC36A1*	GGCTATCGTCACTGCCCTCTA	ACAGTTGGGCAGGTTGAGAGTT	NM_001192498.1
*ZO-1*	TCTGCAGCAATAAAGCAGCATTTC	TTAGGGCACAGCATCGTATCACA	XM_024982009.1
*CLDN4*	TCATCGGCAGCAACATCGTCAC	CAGCAGCGAGTCGTACACCTTG	XM_005892850.2
*OCLN*	GAACGAGAAGCGACTGTATC	CACTGCTGCTGTAATGAGG	NM_001082433.2
*VEGF*	GGAGTTTGGAGCAGCAACAA	TTTGGGGCCTTGAGAGAGAG	NM_174488.2
*NOS3*	TGGATGAGTATGACGTGGTGT	GCGTTTCCAGCTCCGTTTG	XM_024990490.1
*IGF-1*	GCTCTGGCCCACGAGTGGAGA	GCCCTCGATCACCGTGCAGTT	NM_001244612.1
*KEAP1*	GATCTACGTTCTTGGGGGCT	CCAGAGGTCATTCGGGTCAC	NM_001101142.1
*NRF2*	CCCAGTCTTCACTGCTCCTC	TCAGCCAGCTTGTCATTTTG	NM_001011678.2
*HO-1*	GGCAGCAAGGTGCAAGA	GAAGGAAGCCAGCCAAGAG	NM_001014912.1
*NQO1*	CTCTGGCCAATTCAGAGTGG	CAGGATCTGAACTCGGGCAT	NM_001034535.1
*mTOR*	AAACCCAGGTGTGATCAATAATGTC	CATCAACCCATTTCCTCATTTCA	XM_002694043.6
*4EBP1*	ACCAGGATCATCTATGACCGGAA	TGTCCATCTCAAACTGTGACTCT	NM_001077893.2
*P70S6K*	GGAAGAACTGCTGGCTCGGAAG	CATCGTCACGTCCATCTGCTCTATC	NM_205816.1
*β-actin*	TCACCAACTGGGACGACA	GCATACAGGGACAGCACA	NM_173979.3
*GAPDH*	GGGTCATCATCTCTGCACCT	GGTCATAAGTCCCTCCACGA	NM_001034034.2

*CAT*, *Catalase*; *SOD2*, *Superoxide Dismutase 2*; *GSH-px*, *Glutathione peroxidase*; *Bcl-2*, *B-cell lymphoma-2*; *Bax, BCL2--Associated X*; *GLUT1*, *GLUT4*, *Glucose transporter 1, 4*; *SLC36A1*, *Solute carrier family 36*, *member 1*; *ZO-1*, *Zona Occludens 1*; *CLDN4*, *Claudin 4*; *OCLDN*, *Recombinant Occludin*; *VEGF*, *Vascular Endothelial Growth Factor*; *NOS3*, *Nitric oxide synthase 3*; *IGF-1, Insulin-like Growth Factors*; *KEAP1*, *Kelch-like ECH-associated protein 1*; *NRF2*, *Nuclear factor erythroid-2-related factor 2*; *HO-1*, *Heme oxygenase 1*; *NQO1*, *NADPH:Quinone Oxidoreductase 1*; *mTOR*, *Mammalian target of rapamycin*; *4EBP1*, *eIF4E-binding protein 1*; *P70S6K*, *Ribosome S6 protein kinase*; *β-actin*, *beta-Actin*; *GAPDH*, *Glyceraldehyde-3-phosphate dehydrogenase*.

## Data Availability

The data presented in this study are available on request from the authors.
